# Reliability and validity of Chengdu pediatric emergency triage criteria: case study of a single center in China

**DOI:** 10.1186/s12887-023-04072-4

**Published:** 2023-05-19

**Authors:** Yingying Zhao, Liqing He, Juan Hu, Jing Zhao, Xiaolan Yi, Humin Huang

**Affiliations:** 1 Department of Emergency Nursing, West China Second University Hospital, Sichuan University/West China School of Nursing, Sichuan University, Chengdu, Sichuan China; 2 Key Laboratory of Birth Defects and Related Diseases of Women and Children (Sichuan University), Ministry of Education, Chengdu, Sichuan China

**Keywords:** Pediatric emergency department, Triage, Criteria, Reliability, Validity

## Abstract

**Background:**

We aimed to examine the reliability and validity of Chengdu pediatric emergency triage criteria in order to provide a reference for the development of pediatric emergency triage within other hospitals.

**Methods:**

We developed Chengdu pediatric emergency triage criteria based on the conditions/symptom, vital signs, and the Pediatric Early Warning Score system within our hospital using the Delphi method in 2020. The simulation scenario triage and real-life triage which were conducted in our hospital during January - March 2021, and the retrospective study of triage records extracted from our hospital’s health information system in February 2022, were used to measure the agreement in triage decisions between the triage nurses, and between the triage nurses and the expert team.

**Results:**

For the 20 simulation cases, the Kappa value of triage decisions between the triage nurses was 0.6 (95% CI 0.352–0.849), and the Kappa value of triage decisions between the triage nurses and the expert team was 0.73 (95% CI 0.540–0.911). For the 252 cases in the real-life triage, the Kappa value of triage decisions between the triage nurses and the expert team was 0.824 (95% CI 0.680–0.962). For the 20,540 cases selected for the retrospective study of triage records, the Kappa value of triage decisions between the triage nurses was 0.702 (95% CI 0.691–0.713); that between Triage Nurse 1 and the expert team was 0.634 (95% CI 0.623–0.647); and that between Triage Nurse 2 and the expert team was 0.725 (95% CI 0.713–0.736). The overall agreement rate in triage decisions between the triage nurses and the expert team in the simulation scenario triage was 80%; that between the triage nurses and the expert team in the real-life triage was 97.6%; and that between the triage nurses in the retrospective study was 91.9%. In the retrospective study, the agreement rates in triage decisions between Triage Nurse 1 and the expert team, and between Triage Nurse 2 and the expert team, were 88.0% and 92.3%, respectively.

**Conclusion:**

Chengdu pediatric emergency triage criteria that developed within our hospital is reliable and valid, and can promote rapid and effective triage by triage nurses.

## Background

This study concerns emergency departments (EDs), an important aspect of the medical service which is responsible for the treatment of critically ill patients. With continuing social development and increased demand for medical treatment, the number of pediatric ED patients has been increasing in recent years, leading to ED crowding. The results of Fernandez et al. [1] have shown that children account for approximately 40% of the ED visits in the United States and account for 25–30% of accident and emergency visits in the United Kingdom. Beck et al. [2] reported that non-emergency patients accounted for 68.4% among ED visits in Austria. In China, the demand for pediatric emergency services has significantly exceeded the existing resources, and only 20% of ED patients are in real urgent situations [3]. Ham [4] found that ED crowding could lead to longer waiting times for ED treatment, increased dissatisfaction among patients and their family members with ED services, and increase in fatalities and injuries brought about by a rising proportion of patients not being treated in a timely manner. Distinguishing critically ill patients from non-emergency patients, provision of timely and effective medical treatment, and improving treatment efficiency are the key objectives of pediatric emergency triage [5].

Pediatric emergency triage criteria have been widely studied and applied in developed countries such as Canada, United States, Australia, and the United Kingdom. In recent years, pediatric emergency triage has been developing in China. Shen et al. [6] developed pediatric emergency triage criteria in 2018 based on triage criteria for adults. Yang [7] developed the 5-level pediatric emergency triage criteria based on Canadian Triage and Acuity Scale Paediatric Guidelines (PaedCTAS). Hu et al. [8] formulated a 5-level pediatric emergency triage criteria, and a triage process that can be undertaken by two nurses by referring to Emergency Severity Index (ESI), Australasian Triage Scale (ATS) and the Canadian Triage Acuity Scale (CTAS). It has been demonstrated that these criteria have good reliability in clinical practice. However, they cannot be applied directly in our hospital because they were developed in certain geographical regions or in specific hospitals other than our own. By referring to internationally recognized triage criteria, such as ESI, PaedCTAS, and China’s pediatric emergency triage criteria, in 2020 we developed Chengdu pediatric emergency triage criteria based on the conditions/symptom, vital signs, and the Pediatric Early Warning Score (PEWS) system within our hospital using the Delphi method [9]. This study tests the reliability and validity of our criteria in order to provide a reference for the development of pediatric emergency triage within other hospitals.

## Methods

This cross-sectional observational study was conducted at our hospital’s ED during January - December 2021. Our hospital, a tertiary women’s and children’s hospital, has two campuses in Chengdu, China. The ED is set across the two campuses. A total of approximately 300,000 pediatric patients visit our ED per year. Our ED operates 3 shifts per day. The triage nurses work 8-hour shifts. Three triage nurses are on each shift at each campus. Each episode of triage takes approximately 2 min to complete.

### Expert team

The expert team was composed of 10 persons who possess expertise of emergency nursing management, diagnosis and treatment of pediatric ED patients, and emergency nursing. Of them, 3 possessed senior-level professional titles and 7 possessed intermediate-level professional titles; 2 held doctoral degrees and 8 held master’s degrees. The expert team was responsible for explaining the pediatric emergency triage criteria to triage nurses, providing triage training, selecting triage simulation scenarios, and directing a real-life triage.

### Study tools

The three research tools most commonly used by Chinese and foreign researchers to evaluate the reliability and validity of triage criteria were adopted in our study. This study consisted of three stages: simulation scenario triage; real-life triage; and, retrospective study on triage records. Kappa values were used to analyze the agreement in triage decisions between triage nurses, and between the triage nurses and the expert team. Nurses selected for this study satisfied the following criteria: (1) ≥ 5 years’ work experience in the ED; (2) ≥ 3 years of triage experience; (3) good identification ability of critically ill patients with common pediatric diseases; and (4) they had received training on pediatric emergency triage, passed relevant examinations, and could skillfully apply the triage criteria.

### Stage 1 Simulation scenario triage

A total of 20 simulation scenarios were designed following the literature review, interviews with triage nurses, and analysis of typical triage cases. Based on two rounds of expert consultation, the content validity index for the simulation cases was 0.89 and Cronbach’s alpha was 0.9, indicating good validity and reliability for the simulation cases. Triage data include chief complaint, gender, age, consciousness, mental state, and vital signs (body temperature, pulse, and respiration; blood pressure and oxygen saturation were checked for patients aged > 5 years); in addition, for patients transferred from other hospitals as well as re-visiting patients, relevant medical history and examination results were collected. The expert team triaged the simulation cases, and the expert team’s triage decision was considered the model decision. Then, 1 expert played the role of pediatric patient and the other 1 expert played the role of family member. Two triage nurses were selected to independently triage the simulation cases in the ED using Chengdu pediatric emergency triage criteria. The accuracy of the 2 nurses’ triage decisions, and the agreement in triage decisions between the triage nurses, and between them and the expert team, were evaluated. Both nurses held undergraduate qualifications and were supervising nurses, and they had worked at the ED for 18 and 22 years, respectively.

### Stage 2 real-life triage

A one-week clinical trial of Chengdu pediatric emergency triage criteria was conducted in our hospital’s ED in March 2021. A total of 252 pediatric patients visiting our ED during the one-week clinical trial period were selected using convenience sampling. The expert team and ED triage nurses independently triaged the patients using our triage criteria, and the degree of agreement in triage decisions between the clinical triage nurses and the expert team was determined. A total of 5 triage nurses participated in the triage trial. All of them possessed undergraduate qualifications and held supervising nurse job titles, and had worked at the ED for more than 10 years.

### Stage 3 retrospective study of triage records

A retrospective study of triage records was conducted in February 2022. The triage records for the whole of 2021 (including age, gender, chief complaint, medical history, and vital signs) were derived from our hospital’s health information system. A total of 20,540 cases were selected using convenience sampling, with our triage criteria being applied to cases later than March 2021. The sample percentages of cases triaged to levels 1 and 2 were 100% due to the small number of these cases. A total of 1466 level 1 and level 2 cases were included in this study. The sample percentage of level 3 cases was 10% and 1573 level 3 cases were included in this study. The sample percentage of level 4 cases was 10% and 17,519 level 4 cases were included in this study. There were 83 ED triage nurses in our hospital. Of them, 72 (86.7%) possessed undergraduate qualifications, 4 (4.8%) possessed master’s degrees, and 7 (8.4%) possessed junior college diploma; 6 (7.23%) were nurses without professional titles, 41 (49.40%) were nurse practitioners, and 36 (43.37%) were supervising nurses; 29 (34.94%) had worked for 3–5 years, 21 (25.30%) had worked for 6–10 years, 14 (16.87%) had worked for 11–15 years, and 19 (22.89%) had worked for 16 or more years. An expert and the two triage nurses who participated in the simulation scenario triage independently triaged the sampled cases in the retrospective study, and the agreement in triage decisions was determined.

### Triage training

The first round of centralized training and assessment on our triage criteria was provided by the expert team for the triage nurses during January - February 2021. The training comprised point-by-point explanation, case analysis and scenario simulation so that the triage nurses could better understand triage levels and ED zones. The expert team assessed the triage nurses following the training. Then, the expert team provided the second round of training for the 2 triage nurses who would participate in the simulation scenario triage. The expert team then provided the third round of training for the 5 triage nurses selected for the one-week triage trial in March 2021. The fourth round of training and assessment was conducted prior to formal application of the triage criteria to ensure that the triage nurses could recall and apply the triage criteria. During clinical application, the expert team collected and analyzed the triage nurses’ answers to questions concerning the cases in an effort to reduce the bias in triage decision-marking.

### Pediatric emergency triage criteria

Chengdu pediatric emergency triage criteria contain 4 level 1 indicators, 51 level 2 indicators, and 23 level 3 indicators; the maximum waiting time to treatment for all triage levels is as follows: level 1 - immediate treatment; level 2 - within 10 min; level 3 - within 30 min; and, level 4 - within 240 min (Table 1) [9].

**Table 1:** Pediatric emergency triage criteria developed within our hospital

Triage levels	Indicators	Description	Value	Maximum waiting time for treatment
Level 1	Conditions / symptoms (critical)	Sudden cardiac arrest, respiratory arrest; airway obstruction or asphyxia; emergency endotracheal intubation/tracheotomy is required; signs of shock; sudden loss of consciousness; signs of cerebral hernia; life-threatening acute poisoning; precipitous birth (umbilical cord was not cut or Apgar score < 3); complex or multiple trauma; most severe or large burns; ocular trauma with eyeball injury		Immediate
	Vital signs	Temperature (℃) Oxygen saturation (SpO2) AVPU (alert, verbal, pain, unresponsive) scale	≤ 35 or ≥ 41 < 90% U	
	Pediatric Early Warning Score (PEWS) Score	PEWS ≥ 5		
	Other	The triage nurse believed that the patient was encountering a life-threatening situation and requiring emergency care		
Level 2	Conditions/symptoms (high risk)	Chest distress, chest pain, heart palpitations, stable vital signs, high risk or potential risk; status epilepticus; convulsion; diabetic ketoacidosis; acute asthma with stable blood pressure and pulse rate; capillary refill time ≥ 3 s; low reaction to mental state and high level of irritability; hypersomnia (able to wake up; fall asleep without stimuli) with unstable vital signs; newborns with temperature of > 38℃; acute poisoning but does not meet level 1 criteria; sudden change in consciousness; incomplete airway obstruction; esophageal foreign body; severe anemia (no active bleeding) 30–60 g/L; abdominal pain (suspected strangulated intestinal obstruction, incarcerated hernia, intussusception, gastrointestinal perforation, or urinary tract calculi) with the pain score > 6; osteofascial compartment syndrome; active bleeding (epistaxis, hematuria, hematochezia, hemoptysis, or hematemesis) with unstable vital signs		< 10 min
	Vital signs	Pulse rate (beats/min)	*P* > 180 (y < 3 months old); *P* > 160 (3 months old ≤ y < 3 years old); *P* > 140 (3 years old ≤ y < 8 years old); *P* > 100 (y ≥ 8 years old)	
		Respiration rate (breaths/min)	*R* > 50 (y < 3 months old); *R* > 40 (3 months old ≤ y < 3 years old); *R* > 30 (3 years old ≤ y < 8 years old); *R* > 20 (y ≥ 8 years old)	
		SpO2	90–92%	
		Systolic blood pressure	> 130 mmHg (≥ 5 years old) or < 75 mmHg (≥ 5 years old)	
	PEWS score	PEWS = 3–4		
	Other	The triage nurse believed that the patient was at a high-risk situation or potential risk but required no emergency care		
Level 3	Conditions/symptoms	Intermittent epileptic seizures; with a history of hyperpyretic convulsion; foreign body aspiration but no breathing difficulty; dysphagia but no breathing difficulty; mental and behavior disorder; severe vomiting; symptoms of allergic reaction (obvious rashes on the skin and mucous membranes, extensive facial swelling, etc.); hypersomnia (able to wake up; fall asleep without stimuli) with stable vital signs; moderate to severe pain with any cause (score: 4–6); stable newborns; active bleeding (epistaxis, hematuria, hematochezia, hemoptysis, or hematemesis) with stable vital signs; unexplained abdominal distension with mental malaise; mucocutaneous hemorrhage/platelet ≤ 20 × 10^9/L		< 30 min
	Vital signs	Pulse rate (beats/min)	88 < *P* < 180 (y < 3 months old); 80 < *P* < 160 (3 months old ≤ y < 3 years old); 64 < *P* < 140 (3 years old ≤ y < 8 years old); 56 < *P* < 120 (y ≥ 8 years old)	
		Respiration rate (breaths/min)	24 < *R* < 50 (y < 3 months old); 20 < *R* < 40 (3 months old ≤ y < 3 years old); 16 < *R* < 30 (3 years old ≤ y < 8 years old); 14 < *R* < 24 (y ≥ 8 years old)	
	PEWS score	PEWS = 1–2		
	Other	The pediatric patient had acute symptoms and emergency issues		
Level 4	Conditions/symptoms	Vomiting or diarrhea without dehydration; Mild pain		< 240 min
	PEWS score	PEWS = 0		
	Other	Mild or non-urgent condition		

### Statistical methods

The collected data were coded, and SPSS23.0 was used for data analysis. Descriptive analysis of data concerning the accuracy rate of triage nurses’ triage decisions in the simulation scenarios was conducted. Spearman rank correlation was used for correlation analysis. The Kappa test was used to measure the coefficients of agreement among the triage decisions between the triage nurses, and between the triage nurses and the expert team. The Kappa test is a commonly used statistical method for testing the reliability of test data relating to triage tools. A Kappa value of < 0.20 represents poor reliability; a Kappa value of 0.21–0.40 represents fair reliability; a Kappa value of 0.41–0.60 represents moderate reliability, a Kappa value of 0.61–0.80 represents substantial reliability; and a Kappa value of 0.81-1.0 represents excellent reliability.

## Results

### Reliability

#### Reliability of triage criteria in simulation scenarios

The agreement in triage decisions on 20 simulation cases between the 2 triage nurses was tested. The Kappa value of triage decisions between the 2 triage nurses was 0.6 (95% CI 0.352–0.849) and *P* = 0.000, indicating moderate agreement. The Kappa value of triage decisions between the triage nurses and the expert team was 0.73 (95% CI 0.540–0.911) and *P* = 0.000, indicating substantial agreement (Table 2).

**Table 2:** Agreement in triage decisions between triage nurses in simulation scenarios

Numbers of cases triaged by Triage Nurse 1	Numbers of cases triaged by Triage Nurse 2				Total
	Triage level 1	Triage level 2	Triage level 3	Triage level 4	
Triage level 1	4	2	0	0	6
Triage level 2	1	3	2	0	6
Triage level 3	0	0	4	0	4
Triage level 4	0	0	1	3	4
Total	5	5	7	3	20

#### Reliability of triage criteria in real-life triage

The expert team and the 5 triage nurses triaged the same 252 pediatric ED patients. Excellent agreement in triage decisions between the triage nurses and the expert team was identified by a Kappa value of 0.824 (95% CI 0.680–0.962) and *P* = 0.000 (Table 3).

**Table 3:** Agreement in triage decisions between the triage nurses and the expert team in real-life triage

Model triage decisions made by the expert team	Number of cases triaged by the triage nurses				Total	Agreement rates %
	Triage level 1	Triage level 2	Triage level 3	Triage level 4		
Triage level 1	0	0	0	0	0	0
Triage level 2	0	5	0	0	5	100
Triage level 3	0	0	10	0	10	100
Triage level 4	0	0	6	231	237	97.4
Total	0	5	16	231	252	

#### Reliability of triage criteria in retrospective study

One expert and the 2 triage nurses triaged the same 20,540 cases selected for the retrospective study of triage records. Substantial agreement in triage decisions between the 2 triage nurses, between Triage Nurse 1 and the expert team, and between Triage Nurse 2 and the expert team was evidenced by a Kappa value of 0.702 (95% CI 0.691–0.713) and *P* = 0.005 (Table 4), a Kappa value of 0.634 (95% CI 0.623–0.647) and *P* = 0.006 (Table 5), and a Kappa value of 0.725 (95% CI 0.713–0.736) and *P* = 0.006 (Table 6), respectively.

**Table 4:** Agreement in triage decisions between triage nurses in retrospective study of triage records

Numbers of cases triaged by Triage Nurse 1	Numbers of cases triaged by Triage Nurse 2				Total
	Triage level 1	Triage level 2	Triage level 3	Triage level 4	
Triage level 1	81	337	186	1	605
Triage level 2	7	187	781	2	977
Triage level 3	3	35	1357	118	1513
Triage level 4	0	1	186	17,258	17,445
Total	91	560	2510	17,379	20,540

**Table 5:** Agreement in triage decisions between Triage Nurse 1 and the expert team in retrospective study of triage records

The expert team’s triage decisions	Number of cases triaged by Triage nurse 1				Total
	Triage level 1	Triage level 2	Triage level 3	Triage level 4	
Triage level 1	98	302	201	4	605
Triage level 2	2	310	645	20	977
Triage level 3	0	82	1179	252	1513
Triage level 4	0	142	461	16,482	17,445
Total	100	836	2486	17,118	20,540

**Table 6:** Agreement in triage decisions between Triage Nurse 2’s and the expert team in retrospective study of triage records

The expert team’s triage decisions	Number of cases triaged by Triage Nurse 2				Total
	Triage level 1	Triage level 2	Triage level 3	Triage level 4	
Triage level 1	62	21	8	0	91
Triage level 2	26	300	234	0	560
Triage level 3	12	372	1804	322	2510
Triage level 4	0	143	440	16,796	17,379
Total	100	836	2486	17,118	20,540

### Validity

#### Validity of triage criteria in simulation scenarios

Of the 20 simulation cases, the numbers of cases triaged to levels 1, 2, 3, and 4 according to the model triage decisions made by the expert team were 6 (30%), 5 (25%), 6 (30%), and 3 (15%), respectively. The overall agreement rate in triage decisions between the triage nurses and the expert team was 80% (75% for level 1, 70% for level 2, 83.3% for level 3, and 100% for level 4). The underestimated triage cases accounted for 15% and mainly occurred with patients who should have been triaged to level (1) Case 1 had a 100% underestimation rate. The overestimated triage cases accounted for 5% and mainly occurred with patients who should have been triaged to level (2) Cases 6 and 10 were overestimated; their overestimation rates were 50% (Table 7).

**Table 7:** Agreement in triage decisions between the triage nurses and the expert team in simulation scenarios

Model triage decisions made by the expert team	Numbers of cases triaged by the triage nurses				Total
	Level 1	Level 2	Level 3	Level 4	
Level 1	9	3	0	0	12
Level 2	2	7^*^	1	0	10
Level 3	0	1	10	1	12
Level 4	0	0	0	6	6
Total	11	11	11	7	40

#### Validity of triage criteria in real-life triage

The expert team and the 5 triage nurses triaged the same 252 pediatric ED patients. The overall agreement rate in triage decisions between the triage nurses and the expert team was 97.6%, and there were no level 1 patients. The agreement rates in triage decisions on levels 2, 3, and 4 between the triage nurses and the expert team were 100%, 100%, and 97.4%, respectively (Table 3).

#### Validity of triage criteria in retrospective study

One expert and the 2 triage nurses triaged the same 20,540 cases selected for the retrospective study of triage records. The overall agreement rate in triage decisions between the triage nurses was 91.9% (13.4% for level 1, 31.7% for level 2, 77.9% for level 3, and 98.9% for level 4), as shown in Table 4. The overall agreement rate in triage decisions between Triage Nurse 1 and the expert team was 88.0% (16.1% for level 1, 31.7% for level 2, 77.9% for level 3, and 94.5% for level 4), as shown in Table 5. The overall agreement rate in triage decisions between Triage Nurse 2 and the expert team was 92.3% (68.1% for level 1, 53.5% for level 2, 71.9% for level 3, and 96.6% for level 4), as shown in Table 6.

## Discussion

### Necessity of triage criteria development

Onset of pediatric diseases tends to be sudden and seasonal. The peak seasons of pediatric diseases are characterized by large numbers of pediatric patients being admitted to our hospital. Due to the limited capacity of outpatient registration or lack of night outpatient service, the patients who cannot be registered in the outpatient department will enter ED, leading to ED crowing. Our hospital received more than 300,000 pediatric ED patients in 2021, of which only 9.98% were critically ill patients. Nevertheless, due to limited medical resources it was not possible for our service to meet all these patients’ demands - in light of which, there is an urgent need for efficient and sensitive pediatric emergency triage criteria. Such criteria must be applied quickly as pediatric patients are young, tend to cry easily, and possess limited oral expression ability [10]. Pediatric emergency triage criteria should enable nurses to accurately and quickly triage patients into the appropriate priority levels [11, 12], thus ensuring them sufficient medical and nursing care. The pediatric emergency triage criteria developed within our hospital was compiled into the health information system. During clinical triage, the triage nurses input data regarding the patients’ vital signs, chief complaints and other relevant information into the system to complete the triage.

### Reliability

Reliability refers to the degree of agreement obtained from repeat measurement of the study object using a research tool. It can show whether the measurement tool can stably measure the object or variables. The higher degree of agreement in the results, the higher degree of reliability of the tool. If agreement in triage decisions is found when a patient is triaged by different persons using the same triage criteria, this triage criteria can be applied clinically as a disease severity assessment tool [13]. Currently simulation scenario triage, real-life triage and retrospective study are most commonly used in the research of reliability and validity [14–17]. Our study used the three commonly used research tools to measure the agreement in triage decisions between the triage nurses, and that between the triage nurses’ triage decisions and the model triage decisions, the same approach as that of Kottner et al. [18].

The results of the simulation scenario triage have shown that the Kappa value of triage decisions between the triage nurses was 0.6 (95% CI 0.352–0.849), indicating moderate agreement; the Kappa value of triage decisions between the triage nurses and the expert team was 0.73 (95% CI 0.540–0.911), indicating substantial agreement. These results are consistent with those of Zhiting et al. [16]. Our triage criteria showed good reliability in the simulation scenario triage.

A prospective study on the real-life triage cases showed a Kappa value of 0.824 (95% CI 0.680–0.962) in triage decisions between the triage nurses and the expert team, indicating excellent agreement. It should be mentioned that Green et al. [19] adopted prospective real-life situations in their triage reliability research, and that Zachariasse et al. [20] conducted a prospective study on 288,663 patients in their research of the effectiveness of the Manchester Triage Scale. A prospective study set in real-life situations can enable triage nurses to make triage decisions based on sufficient information of chief complaints, vital signs and PEWS scores of the real patients. As mentioned above, the agreement in triage decisions in real-life situations was higher than that in simulation scenarios, possibly because the triage nurses could not obtain sufficient information concerning the patients in the simulation scenarios and they triaged the patients in an enclosed space, which is different from real clinical environment. The results in the real-life triage cases suggested that the triage criteria is clinically reliable, enabling triage nurses to make triage decisions based on evidence, and avoiding empirical triage. However, in the real-life situations in our study, there were no level 1 patients and the number of level 2 patients was small, so it is necessary to expand the sample size in future reliability studies.

Our retrospective study of triage records showed substantial agreement in triage decisions between the triage nurses, between Triage Nurse 1 and the expert team, and between Triage Nurse 2 and the expert team, which are consistent with the results of Wuerz et al. [21] who found a high degree of agreement in triage decisions between triage nurses and physicians in the test of reliability of ESI. The Kappa values of triage decisions between the triage nurses and between the triage nurses and the expert team found in our study are higher than the Kappa value of 0.32 (95% CI 0.24–0.40) reported by Branes et al. [17] for 200 pediatric patients, indicating that Chengdu pediatric emergency triage criteria has a high degree of reliability, possibly because our study has large overall sample size and a large sample size of level 4 patients, which reduced the bias in the results.

### Validity

Validity reflects whether a measurement result is valid or accurate. The validity of triage criteria refers to the accuracy of triage based on the severity and emergency of patients’ conditions [21]. The results of the simulation scenario triage have shown that the overall agreement rate in triage decisions between the two triage nurses and the expert team was 80% (75% for level 1, 70% for level 2, 83.3% for level 3, and 100% for level 4); and the agreement rates in triage decisions on levels 1 and 2 were low. In the simulation scenario triage, 3 patients who should be triaged to level 1 were triaged to level 2, 2 patients who should be triaged to level 2 were triaged to level 1, and 1 patient who should be triaged to level 2 was triaged to level 3. The differences in triage decisions between the triage nurses and the expert team were not large. There were small number of simulation cases in our study. It is suggested that the number of simulation cases should be increased to further analyze its influencing factors. The overall agreement rate in triage decisions between the triage nurses and the expert team in the real-life triage and the agreement rates in triage decisions on levels 2, 3, and 4 are high, which were consistent with the results of Ng et al. [22] in their research of Taiwan triage and acuity scale, showing that Chengdu pediatric emergency triage criteria has good validity.

The results of our retrospective study of triage records showed that the overall agreement rate in triage decisions between triage nurses was 91.9%, but the agreement rates in triage decisions on levels 1 and 2 were 13.4% and 31.7%, respectively; the agreement rates in triage decisions on levels 1 and 2 between Triage Nurse 1 and the expert team were low, but that between Triage Nurse 2 and the expert team were high, possibly because there were different effects of training on the nurses and they had different levels of understanding of the triage criteria; moreover, differences in work experience, professional titles, frequency for training might be associated with the accuracy of triage decisions. It is advisable to provide continuous triage training for training nurses.

### Mistriage

In the simulation scenario triage, the underestimation and overestimation rates were 15% and 5%, respectively, which are lower than that reported by Chen et al. (underestimation rate of 26% and overestimation rate of 12.67%) [14]. Overestimation can lead to wastage of medical resources, and underestimation can lead to delayed treatment in patients who are in real need of emergency care. Since the simulation scenarios were not real-life clinical situations, the triage nurses could not obtain sufficient information concerning the patients in the simulation scenarios. Triage nurses make triage decisions based on the chief complaints, symptoms and signs, and PEWS scores of the patients. The occurrences of underestimation and overestimation in the simulation scenarios may be attributed to the arbitrarily limited range of information, in contrast to that obtained by nurses via the more dynamic interactions with live patients in real-life triage situations. The underestimation mainly occurred with patients who should have been triaged to level (1) The underestimation rate for Case 1 was 100%. In case 1, the patient had a fever and two convulsions within 30 min and was comatose throughout. The underestimation occurred in Case 1 possibly because the triage nurses only evaluated the patient’s vital signs but did not adequately evaluate the patient’s consciousness. Overestimation mainly occurred with patients who should have been triaged to level (2) The overestimation rates in cases 6 and 10 were both 50%. The patient in Case 6 was a child with paroxysmal supraventricular tachycardia. The reason for overestimation might have been that the acute condition of the patient affected the nurses’ triage decision-making. The patient in Case 10 was a child with adverse reactions following intake of cephalospora. One possible reason for the overestimation might be that the triage nurse first considered the possibility of anaphylactic shock caused by the drugs based on clinical experience. Although incidences of overestimation and underestimation occurred in our study, the differences in triage decisions between the triage nurses and the expert team were not large. For example, the patients who should have been triaged to level 1 were triage to level 2, and the patient who should have been triaged to level 2 were triaged to levels 1 or 3, the data suggest that our triage criteria has high degree of agreement and accuracy. However, it is suggested that the number of simulation cases should be increased to further analyze its influencing factors.

The overestimation and underestimation also occurred in our retrospective study, possibly because crying patients gave rise to the difficulty and inaccuracy in the collection of patient information; the patients’ family members gave an exaggerated description on the patients’ conditions in order to see a doctor as soon as possible; or the triage nurse’s high stress caused them to rise the triage level, leading to overestimation.

### Limitations

Although the findings of our study indicate that the reliability and validity of pediatric emergency triage criteria developed within our hospital is acceptable, some problems also exist. First, the sample size of simulation scenario triage cases was small. The agreement in triage decisions between the nurses was moderate. The sample size of the simulation cases should be increased in further studies. Second, our triage criteria has only been clinically applied in our hospital, a national-level hospital with a large number of pediatric patients, and multi-center research is required. Third, the test-retest reliability should be assessed in the retrospective study in order to improve the reliability and validity of the triage criteria.

## Conclusions

This study has determined that Chengdu pediatric emergency triage criteria developed within our hospital has good reliability and validity. Our study adopted triage simulations and real-life triage settings, as well as a retrospective study of collate triage data covering a 12-month period. Analysis of these combined data streams suggest that our triage criteria is reliable and valid and can promote rapid and effective triage by triage nurses. It is hoped that the methods and findings of this study may inform the development of pediatric emergency triage criteria in other hospitals.

## Data Availability

The datasets used and/or analyzed during the current study are not publicly available because the participants’ personal information was included in this study, but the data that were not involved the participants’ personal information are available from the corresponding author on reasonable request.

## Abbreviations

ATS: Australasian Triage Scale
CTAS: Canadian Triage Acuity Scale
ED: Emergency department
ESI: Emergency Severity Index
PaedCTAS: Canadian Triage and Acuity Scale Paediatric Guidelines
PEWS: Pediatric Early Warning Score
